# Visual gravitational motion and the vestibular system in humans

**DOI:** 10.3389/fnint.2013.00101

**Published:** 2013-12-26

**Authors:** Francesco Lacquaniti, Gianfranco Bosco, Iole Indovina, Barbara La Scaleia, Vincenzo Maffei, Alessandro Moscatelli, Myrka Zago

**Affiliations:** ^1^Department of Systems Medicine, University of Rome Tor VergataRome, Italy; ^2^Centre of Space Bio-medicine, University of Rome Tor VergataRome, Italy; ^3^Laboratory of Neuromotor Physiology, IRCCS Santa Lucia FoundationRome, Italy; ^4^Department of Cognitive Neuroscience, University of BielefeldBielefeld, Germany

**Keywords:** internal model, interception, microgravity, time perception, insula, temporo-parietal junction, self-motion

## Abstract

The visual system is poorly sensitive to arbitrary accelerations, but accurately detects the effects of gravity on a target motion. Here we review behavioral and neuroimaging data about the neural mechanisms for dealing with object motion and egomotion under gravity. The results from several experiments show that the visual estimates of a target motion under gravity depend on the combination of a prior of gravity effects with on-line visual signals on target position and velocity. These estimates are affected by vestibular inputs, and are encoded in a visual-vestibular network whose core regions lie within or around the Sylvian fissure, and are represented by the posterior insula/retroinsula/temporo-parietal junction. This network responds both to target motions coherent with gravity and to vestibular caloric stimulation in human fMRI studies. Transient inactivation of the temporo-parietal junction selectively disrupts the interception of targets accelerated by gravity.

## INTRODUCTION

Humans as well as other animals very often experience the vision of objects accelerated by Earth gravity, such as objects in free-fall, projectile, or pendulum motion. Also self-motion may involve an optic flow accelerated by gravity, as when falling or jumping from a height. Whether an object is moving (object-motion), we are moving (self-motion) or both are moving, we must be able to predict the future trajectory of the target to bring about desirable collisions (making interceptions), avoid unwanted collisions or simply anticipate the future course of an event we are watching. Indeed, survival of animals in the forest often depends on accurate estimates of flight time for either self-motion or object motion. Thus, a predator jumping off a tree must time its flight to grab a prey on the spot, while the prey must time the escape from the predator to avoid being caught. Humans are more often engaged in less dangerous but equally demanding tasks, as when they practice sports such as down-hill skiing, trampoline jump or diving, all of which involve gravitational self-motion. Gravitational object motion is experienced, for instance, when we try to save an object which has slipped through our fingers. Also, watching or playing many recreational or sport activities involve the predictive estimate of the movement time of a flying ball.

Predicting the vertical component of target motion under gravity (neglecting air drag) is equivalent to solving the equations:

$$x\left(t+\Delta t\right)=x\left(t\right)+\dot{x}\left(t\right)\Delta t-0.5g\Delta t^{2}$$

$$\dot{x}\left(t+\Delta t\right)=\dot{x}\left(t\right)-g\Delta t$$

*x*(*t*) and ẋ(t)are the vertical position and speed of the target at a given time *t*, while *x*(*t* + Δ*t*) and ẋ(t+Δ t)are the position and speed after a Δ*t* time interval, and *g* is the acceleration due to gravity (about 9.8 m s^-^^2^). In other words, the model equations extrapolate current position and speed of the target Δ*t* in the future. Our brain presumably does not solve the equations explicitly, but it must extrapolate target trajectory one way or another in order to compensate for the intrinsic delays in processing sensory and motor information. Without extrapolation, the neural estimates of position and speed of a visual target at a given instant of time would correspond to values sometime in the near past, and we would intercept or avoid collision at a place where the target used to be, rather than where the target currently is (Nijhawan, 2008).

Delays cumulate as information is processed during the visuomotor transformations leading to a response. Thus, neural responses in the middle-temporal (MT) area of the monkey (a critical region for visual motion processing) lag by about 50 ms behind the changes in target speed (Lisberger and Movshon, 1999; Krekelberg, 2008). It takes at least another 100–150 ms to translate these neural visual signals into an overt motor response (such as that involved in reaching and catching), resulting in a net visuomotor delay of about 150–200 ms (Zago et al., 2008, 2009; Vishton et al., 2010). On-going visual information for a moving target may be updated faster than for the sudden appearance of a stimulus, but overall visuo-motor delays can hardly fall below about 110 ms (Brenner and Smeets, 1997; Zago et al., 2008, 2009).

A correct extrapolation relies on an estimate of the gravitational acceleration *g*. However, the visual system does not have direct access to the absolute *g*, but only to the corresponding retinal image information (Regan, 1997). Whereas *g* is constant at a given location, the acceleration of the resulting image on the retina is not constant at all, but varies inversely with viewing distance. The problem is that the visual system is quite poor at estimating image accelerations (Werkhoven et al., 1992; Dessing and Craig, 2010), as is the oculomotor pursuit system in tracking accelerated targets (Watamaniuk and Heinen, 2003; Bennett and Benguigui, 2013). Nevertheless, visual perception (Moscatelli and Lacquaniti, 2011; Indovina et al., 2013a) and manual interception of targets accelerated by gravity can be very precise (Lacquaniti and Maioli, 1987, 1989; Zago et al., 2004, 2008, 2009; Vishton et al., 2010). It follows that the brain must rely on some trick to supplement on-line visual signals in order to take into account the effects of gravity on object motion or self-motion.

One hypothesis is that the effects of gravity are taken into account by combining multisensory information with *a priori* information about the direction and magnitude of the gravity vector, resulting in an internal model able to predict target motion under gravity (Zago et al., 2004; Indovina et al., 2005; Zago and Lacquaniti, 2005a, c). (An internal model is a neural process that mimics a physical event, see Kawato, 1999; Merfeld et al., 1999) According to this hypothesis, the internal model of gravity effects is used to tune motor responses or perceptual judgments of visual gravitational motion. The vestibular system integrates multisensory information, including vestibular, visual and proprioceptive cues (Fukushima, 1997; Lopez and Blanke, 2011), and represents the prime system for providing gravity-related signals. Here we describe behavioral and neural responses to visual gravitational motion, and we consider putative mechanisms for processing gravity effects on a target motion. Studies of object motion are reviewed first, followed by studies of self-motion.

## OBJECT MOTION

### BEHAVIORAL RESPONSES

There is ample behavioral evidence that Earth’s gravity is taken into account in several forms of implicit knowledge, including visual perception or memory of object-motion. Thus, gravity is taken into account when judging the duration of motion of a falling target (Grealy et al., 2004; Huber and Krist, 2004; Brouwer et al., 2006; Moscatelli and Lacquaniti, 2011). Moreover, the final position of a horizontally moving target (Hubbard, 1995) or a projectile (De Sá Teixeira et al., 2013) that are suddenly halted is misremembered as being displaced downward below the path of motion, consistent with the idea that gravity effects are implicitly assumed by the observers. The oscillations of a pendulum represent another familiar example of gravitational motion. Visual perception is sensitive to deviations from the relation between pendulum period and pendulum length (Bozzi, 1958; Pittenger, 1990; Frick et al., 2005). Indeed, in experiments in which a pendulum oscillates faster or slower than normal, the observers rate the oscillations violating the physical length-period relation less natural than those complying with physics (Pittenger, 1990).

The largest body of evidence for an internal model of gravity effects on target motion has been accumulated in studies of manual interception of a falling object (Zago and Lacquaniti, 2005a,c; Zago et al., 2008, 2009). Depending on the specific protocol, interception could involve catching (Lacquaniti and Maioli, 1987, 1989; Lacquaniti et al., 1993; Vishton et al., 2010), punching (Zago et al., 2004, 2005; Zago and Lacquaniti, 2005b) or batting (Katsumata and Russell, 2012) a ball dropped vertically. In all cases, the movements were well synchronized with the arrival of the ball. In particular, anticipatory electromyographic (EMG) responses in upper limb muscles were roughly time-locked to the expected arrival of the ball, independent of the height of fall when this was changed from trial to trial (Lacquaniti and Maioli, 1987, 1989).

A similar anticipatory activity has been described for manual catching of a ball thrown in projectile motion (Savelsbergh et al., 1992; Cesqui et al., 2012; D’Andola et al., 2013). Gravity effects appear to be taken into account also in the oculomotor behavior necessary to track projectile motion (Diaz et al., 2013). Gómez and López-Moliner (2013) recently showed that knowledge of absolute target size (*s*) and gravity (*g*), combined with signals about optical size of the target (visual angle θ), its elevation angle (*γ*) and time derivative (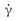), can provide reliable estimates of projectile motion in 3D. The corresponding time-to-contact (TTC) estimate for interception is defined by:

$$TTC=\frac{2s\dot{\gamma }}{g\theta \,\;\cos\gamma }$$

While target size and gravity are constants related to the context, optical size, elevation angle and its time derivative are time-varying variables derived from on-line visual information.

Predictive behavior related to the anticipation of gravity effects has also been revealed by occluding the terminal phase of target motion (Dessing et al., 2009; Zago et al., 2010; Baurès and Hecht, 2011; Bosco et al., 2012; Katsumata and Russell, 2012) or by stopping target motion unexpectedly before arrival (Vishton et al., 2010).

The bulk of the studies cited above show that TTC estimates for motions accelerated by gravity take into account target acceleration. Gravity is such a strong acceleration that estimates neglecting it would lead to considerable timing errors, especially over relatively short heights of target fall (Tresilian, 1999; Zago et al., 2008). This contrasts with many interceptive or avoidance tasks which involve motion not affected by gravity, such as horizontal motion. Horizontal motion is often uniform (at constant speed) or accelerations are so modest to be safely neglected. Indeed, there is much experimental evidence that first-order estimates based on optical variables related to position and velocity are used to accurately predict the TTC for targets moving along the horizontal (Lee, 1976; Tresilian, 1999; Regan and Gray, 2000; Zago et al., 2009). One such optical variable that has received special attention is represented by tau, defined as the ratio between image size and its rate of change (Lee, 1976). Tau can provide a direct estimate of TTC for a target approaching at constant speed the observer along the sightline, with no need to estimate the object’s distance and speed relative to the eye, nor the object’s absolute size.

#### Performance in weightlessness

In contrast with the accurate performance associated with targets accelerated by Earth gravity, the interception performance with targets descending vertically at constant speed (0*g*) is often inaccurate, movements being timed too early. Real 0*g* (weightless) conditions were tested in astronauts during orbital flight (McIntyre et al., 2001), while 0*g*-motion of a visual target was simulated in the laboratory (Zago et al., 2004, 2005; Zago and Lacquaniti, 2005b). The timing errors are striking, because motion at constant speed can be measured reliably by the visual system (McKee et al., 1986; de Bruyn and Orban, 1988; Werkhoven et al., 1992), and first-order TTC estimates are successfully used in case of horizontal motion, as noticed above. Therefore, if subjects relied entirely on visual feedback, with practice they should be able to intercept 0*g* targets descending vertically, just as they do with horizontally moving targets. Instead, the persistence of timing errors observed even after 14 days in orbit is consistent with the operation of an internal model which assumes that descending targets are always accelerated by Earth gravity (Lacquaniti et al., 1993; Tresilian, 1999; McIntyre et al., 2001; Zago and Lacquaniti, 2005b).

#### Role of vestibular signals

A series of studies showed that vestibular signals detecting the direction of gravity can be used to tune motor behavior in response to visual gravitational motion. Senot et al. (2005) asked subjects to intercept a ball approaching in a virtual scene presented stereoscopically in a head-mounted stereoscopic display. Subjects either pitched their head backward so as to look up toward the ball falling from a ceiling, or they pitched their head downward so as to look toward the ball rising from a floor. The visual reference frame for up and down was anchored to the physical gravitational vertical, as sensed by the vestibular system. It was found that subjects were more accurate at intercepting targets whose motion obeys gravity (accelerating while they descend from above and decelerating while they ascend from below), rather than targets whose motion violates gravity (decelerating while descending and accelerating while ascending). This fits with the idea that interception timing depends on gravity-related information (Senot et al., 2005; Le Séac’h et al., 2010). In particular, because otolith sensory organs respond differently according to the orientation of the head with respect to gravity (Fernandez and Goldberg, 1976), they help defining the direction of expected gravity acceleration.

Consistent with this hypothesis, a study performed during a parabolic flight campaign provided evidence for a contribution of otolith sensors in the visuomotor responses to accelerating/decelerating targets (Senot et al., 2012). During each parabola, a 20-s weightless (0*g*) phase is preceded and followed by 20-s of hypergravity (1.5–1.8*g*). The unloading of the otoliths when passing from hypergravity to hypogravity is sensed as a negative gravity, i.e., as a gravitational pull in the upward direction. Strikingly, the timing of the interceptive responses in the virtual environment described above (Senot et al., 2005) reversed sign during the weightless phases compared with the responses at normal gravity (Senot et al., 2012). This reversal, therefore, can be attributed to a corresponding reversal of the otolith responses during the transition from hypergravity to hypogravity.

#### Virtual gravity defined by visual cues

An up/down reference can be strongly biased by contextual cues included in the visual scene. Indeed, as mentioned above, astronauts continued to anticipate the effects of Earth gravity on a ball projected “downward” from the ceiling of the space shuttle (McIntyre et al., 2001). On Earth, the effects of a virtual gravity in a visual scene with strong up/down cues are anticipated even when the target moves in a head-to-feet direction of supine subjects (Miller et al., 2008) or in an oblique direction of seated subjects (Moscatelli and Lacquaniti, 2011).

Not only can pictorial cues affect the perception of gravity direction, but they also contribute mapping between retinal and world information and calibrating the effects of gravity on a visual target by providing a perspective metric (Zago et al., 2009). In order to process visual gravitational motion, the brain must combine target motion, which is represented topographically on the retina, with an internal representation of gravity, which is presumably specified in the world coordinates of the visual scene. This combination requires making reference to a common spatial frame. Retinal motion information might be scaled by the viewing distance to estimate target motion in world coordinates. Eye vergence, accommodation and stereo-disparity may contribute to estimating viewing distance of target motion in 3D space, but these cues are ineffective when the target is far or when it moves on a 2D video display (as in a videogame). Pictorial information such as that provided by natural objects in the visual scene also aids recovery of an environmental reference and scale (Distler et al., 2000). For instance, if an object fell near a person, the estimated height of the person can be used to scale the motion of the falling object, effectively recovering the apparent distance from the viewer (Miller et al., 2008). Indeed, consistent with the idea that pictorial information about the scale of the scene helps calibrating the effects of gravity, when such pictorial information is missing, the interception performance with targets accelerated by gravity is considerably worse than in the presence of pictorial information (Miller et al., 2008).

Zago et al. (2011a) manipulated the alignment of virtual gravity and structural visual cues between each other, and relative to the orientation of the observer and physical gravity. A factorial design assessed the effects of the scene orientation (normal or inverted) and the direction (normal or inverted) of virtual gravity affecting target motion. It was found that interception was significantly more successful when scene direction was concordant with target gravity direction, irrespective of whether both were upright or inverted. These results show that the visible influence of virtual gravity and pictorial cues can outweigh both physical gravity and viewer-centered cues, leading to rely instead on the congruence of the apparent physical forces acting on people and objects in the scene. In another study, it was shown that the presence of biological movements in animate scenes helps processing target kinematics under the ecological conditions of coherence between scene and target gravity directions (Zago et al., 2011b). In this study, button-presses triggered the motion of a bullet, a piston, or a human avatar (animated with actually recorded biological motion) that intercepted the moving target. The timing errors were smaller with the human avatar than the bullet or piston, but only when the directions of scene and target gravity were concordant.

#### Combination of cues

Estimates of the direction of gravity effects on a target motion generally depend on a combination of multiple cues. Such a combination was revealed in the study by Moscatelli and Lacquaniti (2011) who asked observers to judge the duration of motion of a target accelerating in one of four different directions, downward, upward, leftward and rightward relative to a visual scene. Downward motion complied with the gravity constraint, whereas motion in the other directions violated this constraint. Observers watched either a pictorial or a blank scene, while being upright or tilted by 45° relative to the monitor and Earth’s gravity. In another condition, observers were upright and the scene was tilted by 45°. It was found that discrimination precision (inversely related to response variability) was better for downward motion than for the other directions, consistent with the action of visual gravity. However, the difference in precision was not constant across conditions, but was highest when both the observer and the pictorial scene were upright and lowest when the target direction in the non-pictorial scene was tilted by 45° relative to an upright observer. To model the graded behavior across conditions, Moscatelli and Lacquaniti (2011) used a linear combination of the three types of cues experimentally manipulated. They found that pictorial cues accounted for 43% of the overall response, orientation of the observer relative to the physical vertical accounted for 37% of the response, and orientation of target motion relative to the physical vertical accounted for the remaining 20%. Similarly, De Vrijer et al. (2008) suggested an ideal observer model for motion percept based on a linear combination of vestibular and visual cues, each cue being weighed as a function of its reliability.

The relevance of egocentric cues specifying the observer’s orientation is in line with much previous work on the perceptual discrimination of scenes, people and actions (e.g., Troje, 2003; Kushiro et al., 2007; Chang et al., 2010). On the other hand, the substantial contribution of visual references intrinsic to the scene, such as the direction of target motion and the presence of pictorial cues, agrees with the observation that viewing a photograph with strong polarization cues indicating relative up and down directions in the picture can alter the perceived direction of the vertical in the real world (Jenkin et al., 2004).

The ability to discriminate upright objects relative to tilted ones is critical, in so far as upright objects tend to be stable while tilted objects may fall down. Lopez et al. (2009) assessed perceptual judgments of the stability (tendency to fall) of pictures of a human figurine with implied motion. They found combination of cues, because judgments are affected by the picture’s orientation with respect to the physical gravity, the participant’s body, and the pictorial gravity embedded in the figurine for directions that are not concordant with the direction of physical gravity.

In sum, spatial representations for the effects of gravity on a target motion are presumably flexible, and can be biased by different egocentric and allocentric references depending on the context and the available cues. This view agrees with the hypothesis that neural estimates of gravity direction are computed by the Central Nervous System as a Bayesian weighted average of multi-cue information, including vestibular, visual, neck and truncal signals, plus a prior distribution about head and body orientation (Van Beuzekom and Van Gisbergen, 2000; Zupan et al., 2002; MacNeilage et al., 2007; De Vrijer et al., 2008). As far as the vestibular signals are concerned, the otoliths cannot distinguish gravity from linear acceleration (according to Einstein’s Equivalence Principle), but measure specific gravito-inertial force (vector sum of gravity minus linear acceleration). However, the vestibular system is able to estimate the gravity vector in head coordinates by combining signals from otoliths and semicircular canals (Merfeld et al., 1999, 2005). Thus, head orientation relative to gravity can be estimated by integrating the vector cross-product of the estimated angular head velocity (derived from canal inputs) and the direction of gravity (derived from otolith inputs).

### NEURAL SUBSTRATES

The hypothesis that the effects of gravity on a target motion are taken into account by combining multisensory information, including visual and vestibular cues, is supported by neuroimaging studies. Senot et al. (2008) used magneto-encephalography (MEG) during hand catches of a real free-falling ball. MEG revealed the temporal dynamics of activation, by showing that peaks of brain activity are evoked in posterior occipital and lateral parieto-temporal regions about 80–100 ms after ball release, and propagate to sensori-motor cortex in about 40 ms. While MEG affords excellent temporal resolution of the neural events, it lacks the spatial accuracy and resolution necessary to localize the activity peaks at specific brain sites. This spatial localization was provided by a series of fMRI studies that employed computer animations of a target moving up and down along a visual vertical defined by context cues (Indovina et al., 2005; Miller et al., 2008; Maffei et al., 2010). The visual vertical was aligned with the physical vertical in Indovina et al. (2005), while it was orthogonal to it and aligned with the subject’s body in Maffei et al. (2010) and Miller et al. (2008). The target could move under gravity (1*g*, decelerating on the way up and accelerating on the way down) or under artificial, reversed gravity (-1*g*, accelerating going up and decelerating coming down). As expected, the comparison of both types of target motion with a no-motion baseline showed activation in an occipital-temporo-parietal network largely overlapping with the classical dorsal stream for visual motion processing (Orban et al., 2003), including early visual areas (human homologs of monkey V1, V2, V3), hMT/V5+, and intra-parietal sulcus (IPS) areas.

#### Network for object motion under gravity

**FIGURE 1:**
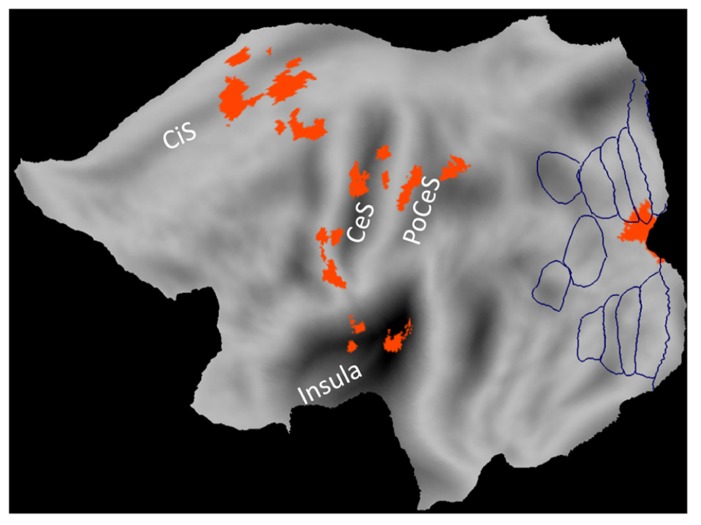
**Brain responses to vertical object motion under gravity.** Statistical parametric maps of the main effect of 1 g motion (data from Indovina et al., 2005) projected on a flat map of the left hemisphere of the human PALS atlas (Caret). Activations correspond to greater blood-oxygen-level-dependent response to vertical motion compatible with gravity (1*g*) than motion incompatible with gravity (-1*g*). Boundaries of visual areas derived from Caret are traced in blue. CeS, central sulcus; CiS, cingulate sulcus; PoCeS, post-central sulcus.

**FIGURE 2:**
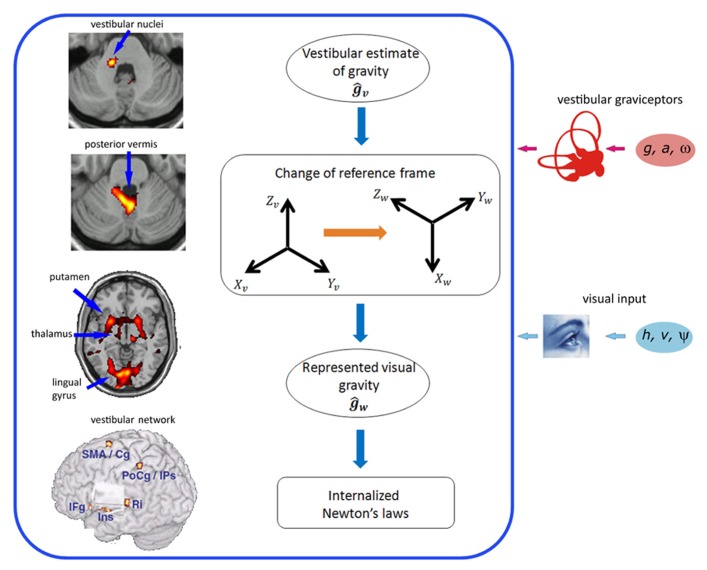
**Brain network (left) and neural computations (right) for processing visual gravitational motion.** Left (top to bottom): activations in vestibular nuclei in the brainstem, posterior cerebellar vermis, putamen, thalamus, lingual gyrus, and overall cortical network of common activations for visual 1*g* motion and caloric vestibular stimulation (peri-sylvian volume removed to show the insular region, deep in the Sylvian fissure). Ins, insula; Ri, retro-insula; IFg, inferior frontal gyrus; PrCg, pre-central gyrus; SMA, supplementary motor area; Cg, middle cingulate gyrus; PoCg, post-central gyrus; IPs, intra-parietal sulcus; SMg, supramarginal gyrus; STg, superior temporal gyrus. Right (top to bottom): The vestibular semicircular canals measure the angular velocity of the head (*ω*), while the otolith organs measure both gravity (*g*) and linear acceleration of the head (*a*). Internal model calculations are included within the box. A vestibular estimate of gravity (*ĝ*_v_) is computed in head-fixed coordinates (*X*_v_, *Y*_v_, *Z*_v_) by the Central Nervous System. Rotational optokinetic cues (*ψ*) and extra-vestibular graviceptive cues may also contribute toward computing *ĝ*_v_. An abstract representation of gravity (*ĝ*_w_) accessible by the visual system is constructed by a change of reference frame to world-fixed coordinates (*X*_w_, *Y*_w_, *Z*_w_), so that it matches the perceived top-bottom axis (*Z*_w_) of the visual scene. The internal model of Newton’s laws results from the combination of *ĝ*_w_ with on-line visual estimates about target motion (*h* and *v* are the vertical position and velocity of the target, respectively), and can be used by the brain for different scopes, such as predicting target TTC, or perceiving a motion as natural. fMRI data in the left are modified with permission from Miller et al. (2008) and Indovina et al. (2005). Neural computations are modified with permission from Indovina et al. (2005).

In the fMRI studies listed above, 1*g* (natural gravity) trials were associated with significantly greater activity than -1*g* (reversed gravity) trials in a network of regions located within and around the Sylvian fissure close to the temporo-parietal junction (TPJ): posterior insular cortex, retro-insula, parietal operculum, supramarginal gyrus, temporal operculum, superior and middle temporal gyri (**Figure 1**). In addition, 1*g* trials engaged sensorimotor cortex including primary somatosensory and motor cortex, ventral premotor cortex, SMA, cingulate cortex, visual cortex including the lingual gyrus, and several subcortical structures including posterior thalamus, putamen, cerebellum and vestibular nuclei (**Figures 1, 2**).

**FIGURE 3:**
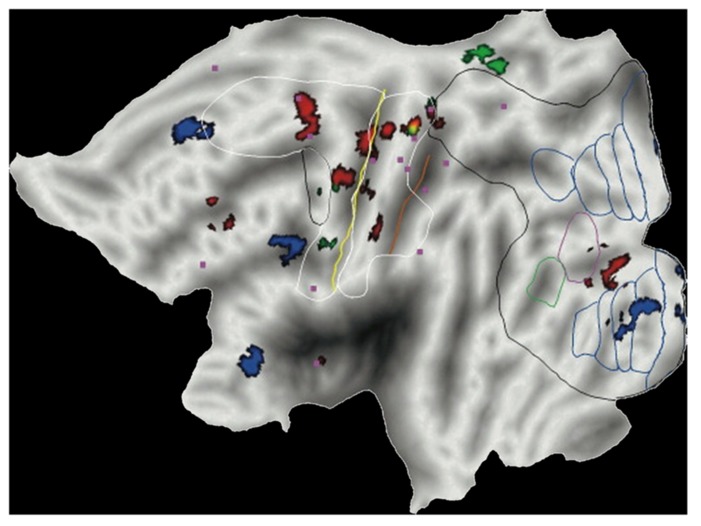
**Brain responses in time-to-contact estimates unrelated to gravity.** In the TTC task, observers decided which of two approaching objects would arrive first. In the inflation task IJ, observers judged which object was expanding faster. In the gap closure task GC, observers judged which of two remote objects translating in the frontoparallel plane would arrive first at a central target location. Activation for the contrast TTC – IJ is shown in red, GC – IJ in green, and IJ – TTC in blue (Reproduced with permission from Field and Wann, 2005.)

An involvement of sensorimotor cortex, SMA, basal ganglia and cerebellum may not be specific of gravity-related motion, but may depend on the temporal prediction of a forthcoming collision, which is more accurate for 1*g* than -1*g* trials. Indeed, a similar engagement of some of these regions is observed in tasks which require perceptual judgments of TTC of targets moving at constant speed, perhaps based on the optical variable tau (Field and Wann, 2005, **Figure 3**). In the monkey, neural discharge in area 7a of the parietal cortex and in primary motor cortex is related to various parameters of stimulus motion, including TTC based on first-order optical cues (Merchant et al., 2004; Merchant et al., 2009).

Instead, the involvement of peri-Sylvian regions close to TPJ appears to be specific of object motion under gravity. Moreover, the neural preference for visual gravitational motion in these regions holds irrespective of the specific spatio-temporal properties of the visual stimulus. Maffei et al. (2010) asked subjects to intercept 1*g* and -1*g* targets either in smooth motion or in long-range apparent motion (LAM, Braddick, 1980). LAM was generated by flashing stationary targets in sequence at different locations along the vertical path, with a wide spatial and temporal separation. Both the insula and lingual gyrus were significantly more active during 1*g* than during -1*g* trials in both real and apparent motion conditions. A region in the inferior parietal lobule showed a preference for 1*g* only during apparent motion but not real motion.

**FIGURE 4:**
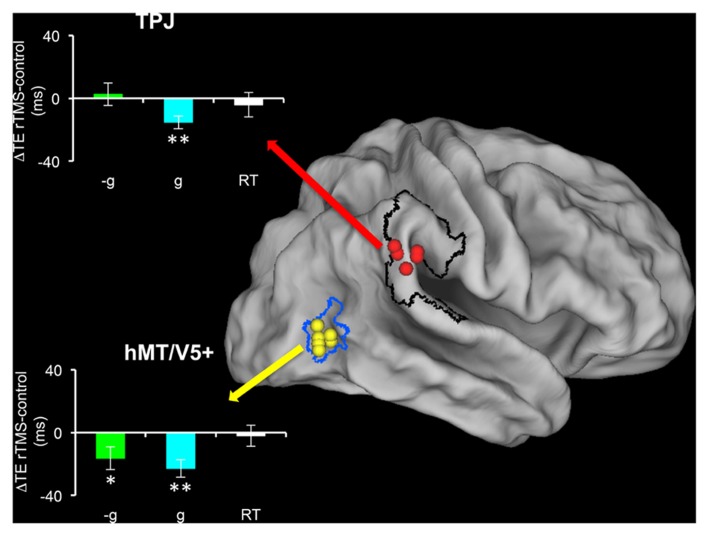
**Effects of repetitive transcranial magnetic stimulation (rTMS) of TPJ and hMT/V5+ on the interception of targets descending along the vertical with natural (1*g*) or artificial (-1*g*) acceleration (modified with permission from Bosco et al., 2008).** Individual TMS sites in TPJ (red) and hMT/V5+ (yellow) are mapped on the Caret PALS human brain (slightly inflated). hMT/V5+ borders (blue) are derived from the probabilistic map of Malikovic et al. (2007), while the black contour delimits the perisylvian region (including TPJ) activated by vestibular caloric stimulation in Indovina et al. (2005). Bar graphs show the mean timing differences (±SEM) between post-rTMS and pre-rTMS interceptive responses. Cyan, 1*g* targets; green, –1*g* targets; white, simple reaction time task, which controlled for specificity of rTMS effects. *p < 0.05; **p < 0.001 (repeated-measures ANOVA).

Bosco et al. (2008) transiently disrupted the activity of TPJ or hMT/V5+ by means of trans-cranial magnetic stimulation (TMS), while subjects pressed a button to intercept targets moving at 1 or -1*g* in the vertical or horizontal direction. They found that TMS of hMT/V5+ affected the interception timing for all tested motion types, whereas TMS of TPJ affected only the interception timing of motion coherent with gravity, that is 1*g* vertical motion (**Figure 4**). Thus, TMS perturbations showed a causal relationship between the activity of TPJ and the processing of visual gravitational motion.

We mentioned above that pictorial information provided by natural objects in the visual scene helps recovering an environmental reference and scale. An fMRI study (Miller et al., 2008) revealed correlates of these visual context effects on gravitational motion processing at a surprisingly early stage of visual-vestibular processing, that is, in the vestibular nuclei and posterior cerebellar vermis (**Figure 2**). In sum, the studies reviewed above indicate that the effects of gravity on object motion are represented in a highly distributed cortical-subcortical network. In a following section, we will show that a similar distributed network underlies the processing of gravity effects during self-motion.

#### Co-localization with the vestibular network

**FIGURE 5:**
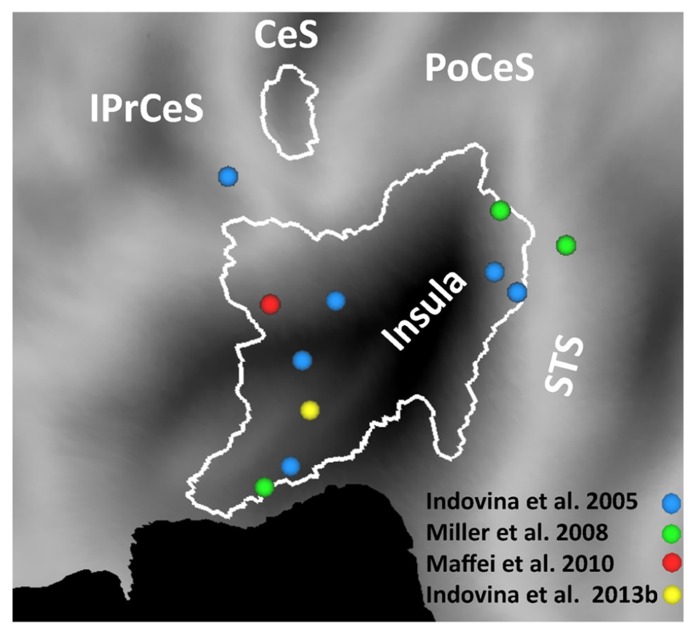
**Brain responses to visual gravitational motion and responses to vestibular stimuli projected on a flat map of the left hemisphere of the human PALS atlas (Caret) to show activations in the Sylvian fissure.** Colored dots denote peaks of activity measured in fMRI studies of visual object motion (cyan, Indovina et al., 2005; green, Miller et al., 2008; red, Maffei et al., 2010) and self-motion (yellow, Indovina et al., 2013b). The peaks identify brain sites showing significantly greater blood-oxygen-level-dependent response to vertical motion compatible with gravity than motion incompatible with gravity. White contours demarcate the borders of regions identified by means of meta-analysis of vestibular caloric studies (with permission from Lopez et al., 2012). CeS, central sulcus; IPrCeS, inferior pre-central sulcus; PoCeS, post-central sulcus; STS, superior temporal sulcus.

Indovina et al. (2005) found that several of the brain sites responding to 1*g* visual stimuli co-localized with the regions independently activated by vestibular caloric stimuli. They then concluded that these regions were presumably identifiable as belonging to the multi-modal visual-vestibular network (**Figure 5**). In fact, posterior insula, retroinsular cortex, and parietal operculum at TPJ possibly represent the human functional equivalent (Brandt and Dieterich, 1999) of the parieto-insular vestibular cortex of the monkey, the core region of vestibular cortex described by Guldin and Grüsser (1998). Indeed, a meta-analysis of 16 human neuroimaging studies using caloric, galvanic, or acoustic stimulation of vestibular receptors shows activation of these regions (Lopez et al., 2012). This meta-analysis was based on a robust activation-likelihood-estimation. The largest clusters of activation were found in the Sylvian fissure, at the level of the insula and retroinsular region, as well as at the temporal and parietal banks of the Sylvian fissure (Lopez et al., 2012; see also zu Eulenburg et al., 2012). The borders of the regions activated by vestibular caloric stimuli derived from the meta-analysis are plotted in the flat map of **Figure 5**. It can be seen that several foci of activation reported in different studies in response to visual gravitational motion (colored dots in **Figure 5**) fall within these borders.

Notice that several of the regions which respond to vestibular stimuli are truly multimodal, because they also respond to optic flow and neck proprioceptive stimuli in human neuroimaging studies (Bense et al., 2001; Bottini et al., 2001; de Waele et al., 2001; Dieterich et al., 2003a,b). Vestibular cortical regions receive di-synaptic inputs from the vestibular nuclei complex via the posterior thalamus (Guldin and Grüsser, 1998; de Waele et al., 2001; Lopez and Blanke, 2011). Lesions of vestibular cortex can lead to a tilt of the perceived visual vertical and rotational vertigo/unsteadiness (Brandt and Dieterich, 1999). A recent clinical report shows that lesions restricted to the posterior insular cortex do not involve vestibular deficits, suggesting that these lesions have to be combined with lesions of adjacent regions of the cortical and subcortical vestibular network to cause vestibular otolith deficits (Baier et al., 2013). Focal electrical stimulation or epileptic discharges around TPJ can elicit sensations of self-motion or altered gravity (Blanke et al., 2002; Isnard et al., 2004; Nguyen et al., 2009). In the monkey, in addition to the vestibular cortex (Guldin and Grüsser, 1998), early visual areas (V2 and V3/V3a) show combined effects of visual and otolith information (Sauvan and Peterhans, 1999). These visual areas might be a functional homolog of the site in the lingual gyrus that is activated by 1*g* trials in human fMRI (Maffei et al., 2010).

## SELF-MOTION

### BEHAVIORAL RESPONSES

Visual perception of heading direction during self-motion relies on multiple cues, including optic flow, monocular or stereo depth, and path (e.g., Duffy and Wurtz, 1991; Warren, 2006; Merchant et al., 2009). The visual effects of gravity may also contribute to heading perception. Vidal et al. (2006) tested the ability to perceive and remember self-motion when subjects are driven passively at constant speed through virtual 3D tunnels that curve in different directions (up, down, left, right). When subjects indicated the amplitude of the turn, they showed a significant asymmetry in pitch-induced perception: downward stimuli produced a stronger pitch perception than upward stimuli, while leftward and rightward yaw turns were perceived equally (Vidal et al., 2006). A subsequent study with the same protocol performed during long-duration space flight aboard the International Space Station showed that weightlessness alters up/down asymmetries in the perception of self-motion (De Saedeleer et al., 2013). Vestibular versus haptic cues were manipulated by having cosmonauts perform the task either in a rigidly fixed posture with respect to the space station or during free-floating. The asymmetry between downward and upward pitch turns observed on Earth showed an immediate reduction when the cosmonauts were free-floating, and a delayed reduction when they were firmly in contact with the floor of the station. Thus, the lack of graviceptive inputs in weightlessness alters the processing underlying the visual perception of self-motion. The finding that the effects on pitch perception are partially overcome by haptic cues indicates the fusion of multisensory (visual, tactile, proprioceptive) cues and top-down cognitive influences.

A different issue concerns the role of visual kinematics during self-motion along the cardinal directions, horizontal and vertical. These directions are typically cued by the orientation of several features of the scene, such as the horizon, trees, buildings, or people. Moreover, kinematics often differs between vertical and horizontal self-motion. Thus, during steady motion, we are typically displaced horizontally at a roughly constant speed, whereas we fall downward and move upward under gravity in an accelerated and decelerated manner, respectively.

Visual estimates of time-to-passage during passive self-motion along the cardinal directions have been reported by Indovina et al. (2013a). Subjects experienced virtual rides on a roller-coaster in a first-person perspective compatible with forward self-motion (Baumgartner et al., 2008). The car traveled along tracks consisting of separate vertical and horizontal rectilinear sections, connected by curves. In both vertical and horizontal sections, the car accelerated, decelerated, or moved at constant speed. Car acceleration/deceleration was coherent with gravity for vertical motion, while the same acceleration/deceleration was rather artificial for horizontal motion. These visual stimuli provide an immersive sense of presence in the virtual environment (Baumgartner et al., 2008), and elicit comparable self-motion sensations across vertical and horizontal paths (Indovina et al., 2013a,b). Subjects were required to press a button when they thought the rollercoaster would pass through a reference point in the scene. In a separate experiment, no visual information was provided during the final part of the path to eliminate the possibility of response triggering upon detection of a given proximity to the target. It was found that, for both visible and occluded conditions, acceleration (positive or negative) was taken into account, but was somewhat overestimated in the calculation of time-to-passage, independently of orientation. Moreover, observers signaled time-to-passage earlier when the rollercoaster accelerated downward at 1*g* (as during free fall), with respect to when the same acceleration occurred along the horizontal orientation. This time shift indicates an influence of the orientation relative to visual gravity due to the anticipation of the effects of gravity on self-motion along the vertical, but not the horizontal orientation. During vertical self-motion, the precision in time-to-passage estimates was higher during accelerated falls than when traveling at constant speed, consistent with a lower noise in time-to-passage estimates when the motion complies with the gravity constraint as compared to when the motion violates the constraint.

### NEURAL SUBSTRATES

**FIGURE 6:**
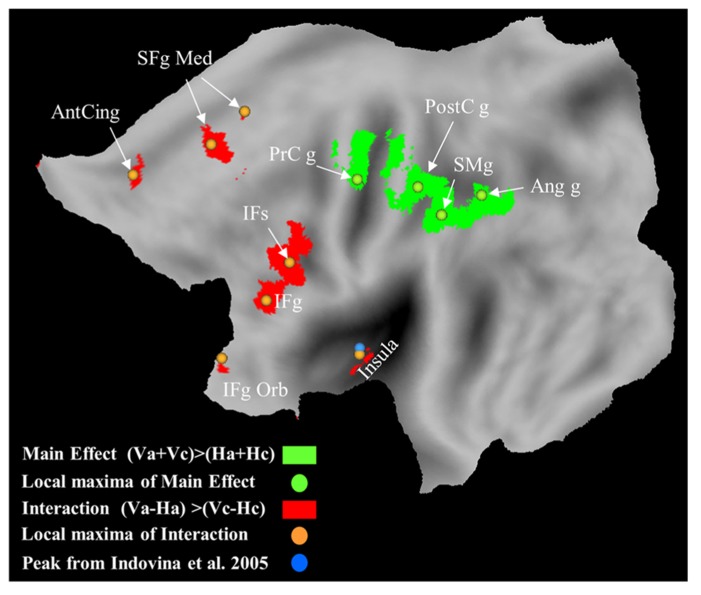
**Brain responses with vertical self-motion compatible with gravity, and with vertical motion independent of motion law.** Statistical parametric maps for the interaction of motion direction by motion law, and maps for the main effect of vertical motion direction are plotted in red and green, respectively. Orange and green dots represent the local maxima for the interaction and main effect, respectively. Cyan dot represents the average maximum in the left posterior insula for vertical object motion coherent with gravity (Indovina et al., 2005). Ant Cing g, anterior cingulate gyrus; IFg, inferior frontal gyrus; IFg Orb, inferior frontal gyrus pars orbitalis; IFs, inferior frontal sulcus; PrCg, pre-central gyrus; PostCg, post-central gyrus; SFg Med, superior frontal gyrus medial; SMg, supra-marginal gyrus.

The neural correlates of passive self-motion in the rollercoaster have been investigated by Indovina et al. (2013b) by using fMRI. Vertical self-motion coherent with gravity engaged the posterior insula, ventral premotor cortex, pre-SMA, cingulate cortex, thalamus, dorsal striatum, cerebellar cortex, and vermis **Figure 6**). These brain regions, but most systematically the posterior insula, have been previously associated with vertical object motion under gravity (Indovina et al., 2005; Miller et al., 2008; Maffei et al., 2010). During self-motion, the retina is specifically activated by the optic flow, and these inputs related to the directional velocity of the image on the retina are relayed via the nuclei of the optic tract and reticularis tegmenti pontis to the vestibular nuclei and the cerebellum and then forwarded to the vestibular cortical network where processing related to the self-motion percept probably occurs.

In the experiments by Indovina et al. (2013b), gravity-related visual kinematics could be extracted from motion signals, by matching the stimuli with a reference gravity template. However, the activation of the posterior insula did not depend on optic flow imbalance between different kinematics. Indeed, it was observed also in a separate experiment where all visual cues (including optic flow) were identical between vertical and horizontal sections. This was obtained by presenting rectilinear motion within dark tunnels, whose direction was cued only by the preceding open-air curves.

Previous fMRI studies reported inconsistent responses of the insula and TPJ (including the retroinsula) to optic flow, with either activations (Antal et al., 2008; Cardin and Smith, 2010) or deactivations (Brandt et al., 1998; Kleinschmidt et al., 2002). Moreover, in a study using 3D vestibular and optic flow stimulation in the monkey (Chen et al., 2010), neurons in the parieto-insular vestibular cortex exhibited robust vestibular responses to both translational and rotational stimuli, but did not respond to optic flow stimulation. Most neurons responding to both sinusoidal rotations and translations are located in the retroinsular cortex. A convergence of signals from the semicircular canals and otoliths in this region as well as the transitional zone with the insular granular field may help disambiguating gravito-inertial forces (see above). Remarkably, a similar convergence could exist also in the human retroinsular cortex, as suggested by the fact that this region is activated by caloric, galvanic and sound stimuli (Lopez et al., 2012).

However, convergence of visual and vestibular inputs related to egomotion has been shown to occur in the monkey visual posterior sylvian area (VPS), which is strongly interconnected to parieto-insular vestibular cortex, as well as in the ventral intraparietal cortex (VIP, Chen et al., 2011a,b). Thus, visual motion regions (such as hMT/V5+, VIP, V6, VPS, and cingulate sulcus visual area) may provide routes for optic flow signals (Smith et al., 2012) to regions such as the posterior insula and the other regions selective for vertical gravitational motion.

**FIGURE 7:**
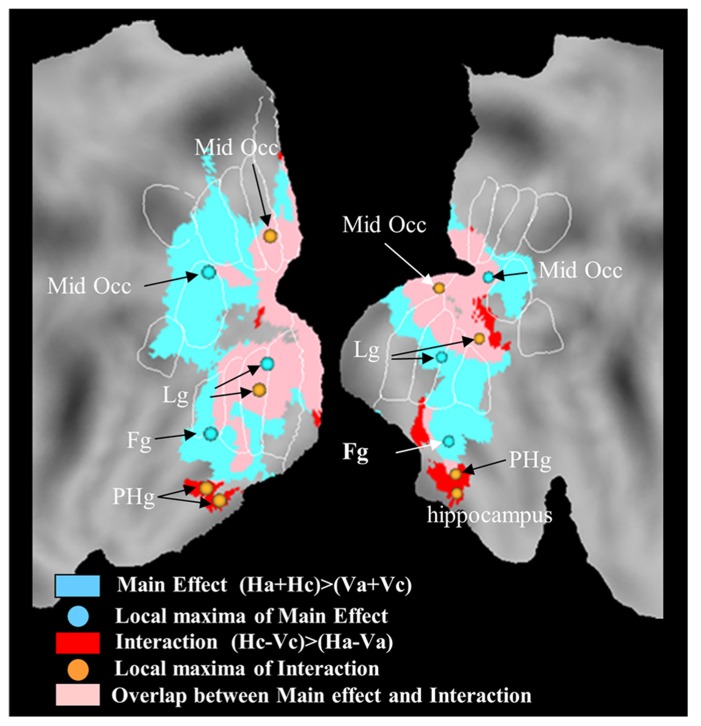
**Brain responses with horizontal self-motion at constant speed, and with horizontal motion independent of motion law.** Statistical parametric maps for the regions exclusively associated with the interaction of motion direction by motion law are plotted in red, those exclusively associated with the main effect of horizontal motion direction in cyan, and those associated with both the main and the interaction effect in pink. Orange and cyan dots represent the local maxima for the interaction and main effect, respectively. Activations are projected onto flat maps of the left (LH) and right (RH) hemisphere of the human PALS atlas (Caret). Fg, fusiform gyrus; Lg, lingual gyrus; Mid Occ, middle occipital gyrus; PHg, para-hippocampal gyrus.

The study by Indovina et al. (2013b) further suggested that neural representations of horizontal self-motion are distinct relative to those of vertical self-motion. In fact, unlike vertical motion, horizontal motion engaged medial-temporal regions including para-hippocampus and hippocampus, consistent with their role in inertial navigation (**Figure 7**).

## CONCLUSION

The evidence reviewed above indicates that the visual effects of gravity are taken into account when dealing with both object motion and self-motion. Perceptual judgments as well as motor interactions with targets accelerated by gravity are much more precise than when the targets move with arbitrary accelerations lacking ecological significance. Because the visual system is poorly sensitive to image acceleration, the most likely explanation for how the brain accounts for gravity effects is that it has internalized them.

The internal model can predict target motion under gravity by extrapolating current information about target position and speed into the future. Occlusion studies show that extrapolation can extend well beyond 1 s durations (Baurès and Hecht, 2011; Bosco et al., 2012). However, the neural model does not solve the motion equations exactly, but provides only an approximate estimate of the trajectory. Estimates become quite accurate and precise in the presence of on-line visual feedback, which tends to correct errors arising from imprecision in the model (Zago et al., 2004). Instead, in the absence of visual feedback, timing errors can be substantial (Senot et al., 2005; Zago et al., 2010; Baurès and Hecht, 2011).

The internal model can be construed as a prior expectation about the underlying forces which act on a target. This prior is normally combined with multisensory information, including visual, vestibular, tactile, and proprioceptive cues. The combination may comply with Bayes’ law, so that robust sensory evidence for the lack of gravitational acceleration can overrule the prior expectation of Earth gravity, especially when context cues about gravity effects are lacking (Zago et al., 2004, 2010). Formally, the prior is a random variable with the following distribution:

$${\hat{g}}_{prior}\approx N\left(9.81,\sigma^{2}\right)$$

The mean of the distribution would be equal to Earth gravitational acceleration, and the variance parameter would account for the variability in the estimate. In Bayesian terms, the posterior estimate is obtained by combining a noisy sensory measurement ĝ_likelihood_ with the prior:

$${\hat{g}}_{posterior\,\;\alpha \,\;}{\hat{\;g}}_{likelihood\,}\cdot {\hat{g}}_{prior}$$

Each term of the second member is weighed inversely to its variance, which measures its reliability. Following a Bayesian interpretation, one would argue that, when the variance in the prior of 1*g* acceleration is very small compared with the variance in the sensory likelihood, the prior prevails, as would be the case of Spacelab experiments or of adaptation experiments with simulated 0*g* targets and context cues about gravity effects (Zago et al., 2005). In other instances, however, the variance in the prior would be large so that sensory evidence prevails, as would be the case when context cues about gravity effects are weak or absent (Zago et al., 2004). Further experiments involving long-term full immersion in reduced gravity environments are needed to validate the Bayesian hypothesis.

In line of principle, the visual effects of gravity (Calderone and Kaiser, 1989) might be dealt with by the brain independently of vestibular signals. This is because, in contrast with the physical gravity which affects the vestibular receptors, visual gravity effects are not invariant but scale with viewing distance. Moreover, visual gravity may not even be aligned with physical gravity, as when we watch a remote scene on a tilted monitor or in weightlessness. However, there is evidence that vestibular signals modulate behavioral responses to visual gravitational acceleration as shown both on Earth (Senot et al., 2005) and parabolic flight (Senot et al., 2012). Moreover, fMRI experiments showed that several of the neural sites responding to visual gravitational acceleration co-localize with the brain regions responding to direct vestibular stimuli (Indovina et al., 2005). TMS experiments further showed that transient inactivation of TPJ, a key region of the cortical vestibular network, selectively disrupts interception of targets accelerated by gravity (Bosco et al., 2008).

To account for these results, it has been suggested that visual processing of targets accelerated by gravity shares the representation of gravity with the vestibular system (Indovina et al., 2005; Zago and Lacquaniti, 2005c). As we remarked above, a posteriori estimates of gravity orientation and effects would derive by a combination of prior information with visual, vestibular, tactile and proprioceptive cues. We now argue that this combination occurs in a network of regions widely distributed in the brain. **Figure 2** (right panels) presents a conceptual scheme illustrating the neural computations which are hypothetically involved in processing visual gravitational motion. According to this hypothesis, the internal model estimating the effects of gravity on seen objects is constructed by transforming the vestibular estimates of mechanical gravity, which are computed in the brainstem and cerebellum, into internalized estimates of virtual gravity, which are memorized in the vestibular network, including cortical and subcortical regions. The integration of the internal model of gravity with on-line visual signals likely takes place at multiple levels in the cortex. This integration presumably involves recurrent connections between early visual areas engaged in the analysis of spatio-temporal features of the visual stimuli and higher visual areas in temporo-parietal-insular regions involved in multisensory integration. Similarly, also the integration with vestibular, tactile and proprioceptive cues occurs in a distributed brain network.

## Conflict of Interest Statement

The authors declare that the research was conducted in the absence of any commercial or financial relationships that could be construed as a potential conflict of interest.
